# Evaluation of Clinical Features and Olfactory Functions in COVID-19: A Multicentre Study

**DOI:** 10.7759/cureus.40027

**Published:** 2023-06-06

**Authors:** Burak Mustafa Taş, Turgay Alpaydın, Sinem Akçalı, Sedat Kaygusuz, Özlem Özlük Erol, Ziya Şencan, Ela Cömert, Nuray Bayar Muluk, Gökçe Özel

**Affiliations:** 1 Otolaryngology, Kirikkale University School of Medicine, Kirikkale, TUR; 2 Anesthesiology Clinic, Manisa Merkezefendi State Hospital, Manisa, TUR; 3 Medical Microbiology, Celal Bayar University Faculty of Medicine, Manisa, TUR; 4 Infectious Diseases and Clinical Microbiology, Kırıkkale University School of Medicine, Kırıkkale, TUR; 5 Infectious Diseases and Clinical Microbiology, Kırıkkale Yüksek Ihtisas Hospital, Kırıkkale, TUR; 6 Otorhinolaryngology, Head and Neck Surgery, Kırıkkale University School of Medicine, Kırıkkale, TUR; 7 Ear Nose Throat, Kirikkale University School of Medicine, Kırıkkale, TUR; 8 Otorhinolaryngology, Gökçe ÖZEL Clinic, Ankara, TUR

**Keywords:** saturation, olfactory dysfunction, covid-19, bsit, bmi

## Abstract

Introduction

Coronavirus Disease-2019 (COVID-19) causes olfactory loss one of the initial diagnostic criteria. The brief smell identification test (BSIT) is an objective test frequently used in olfactory dysfunction. This study aimed to observe the changes in olfactory functions and clinical features in a short time in COVID-19.

Methods

In this prospective study involving 64 patients, the BSIT was performed at two different times; at the time of first application and on the 14th day. Demographic features, laboratory findings, body mass index (BMI), blood oxygen saturation values (SpO2), complaints at first admission, fever, follow-up place, and treatment schemes were noted.

Results

There was a significant difference between the BSIT scores at the first admission and when the polymerase chain reaction (PCR) became negative on the 14th day (p<0.001). Low oxygen saturation values at first admission were associated with low BSIT scores. No relationship was found between olfactory functions and complaints at admission, fever, follow-up place, and treatment schemes.

Conclusion

As a result, negative effects of COVID-19 on olfactory functions have been demonstrated even in the short follow-up period. In addition, low saturation values at first admission were associated with low BSIT scores.

## Introduction

Severe Acute Respiratory Syndrome-Coronavirus-2 (SARS-CoV-2) is a new type of coronavirus responsible for the Coronavirus Disease-2019 (COVID-19) pandemic. After spreading from China to the whole world, the COVID-19 pandemic was declared by the World Health Organization (WHO) on March 11, 2020. In addition to the nonspecific symptoms accompanying viral infections, smell, and taste disorders are very common in COVID-19 patients [1]. It has been reported that at least two of the complaints of smell and taste disorders, nasal congestion, nasal discharge, and fever are observed even in mildly symptomatic patients [2]. Since the beginning of the COVID-19 pandemic, numerous studies have been conducted on presenting complaints and clinical features [3,4].

The incidence of olfactory dysfunction due to COVID-19 can be seen up to 85.6% [5]. Various subjective and objective tests have been used to detect olfactory dysfunction [6]. The brief smell identification test (BSIT) is one of the frequently used objective tests developed from the University of Pennsylvania smell identification test (UPSIT) [7,8].

The present study aimed to examine the clinical features and olfactory functions evaluated with BSIT in patients with positive reverse transcription polymerase chain reaction (rRT-PCR) test in the first 24 hours and when the PCR became negative on the 14th day.

## Materials and methods

This study was carried out with the approval of the Kırıkkale University Scientific Research and Publication Ethics Committee (Decision No: 08/01). Informed consent was obtained from all patients adhering to the Helsinki Declaration. The study was conducted prospectively between August 2020 and March 2021. It was carried out in three centers, Kırıkkale University Medical Faculty Hospital, Kırıkkale Yüksek Ihtisas State Hospital, and Manisa Merkezefendi State Hospital (all three hospitals are referral centers), 67 patients were included in the study, but three patients were excluded due to PCR positivity lasting longer than 14 days. Patients who were diagnosed with COVID-19 within 24 hours at the latest and were followed outside the intensive care units were included in the study. The age, gender, and body mass index (BMI) of all the patients were noted. BSIT scores, blood oxygen saturation (SpO2) values, white blood cell (WBC), hemoglobin, platelet, ferritin, and C-reactive protein (CRP) values at the first admission (when the patient became PCR positive) were obtained, and repeated when the PCR became negative on the 14th day. During the epidemic period when the study was conducted, PCR was used to check whether the disease became negative on the 14th day. Therefore, in our study, the second PCR test was performed on the 14th day.

Inclusion criteria were determined as patients aged 18-60 years and diagnosed with COVID-19 for the first time. Patients were requested to be included in the study within 24 hours of diagnosis. Exclusion criteria were being younger than 18 years of age and over 60 years of age, having PCR positivity lasting more than 14 days, having previous nasal surgery, having major nasal pathology (such as allergic rhinitis, acute-chronic sinusitis, tumor), being hospitalized in the intensive care unit, head and neck oncological diseases and trauma history.

Fever was accepted as 37.5^o^C and above. Patients' complaints at first admission were classified as fever, cough, fever+cough, fever+cough+dyspnea, cough+dyspnea, runny nose, and olfactory dysfunction. Patients were classified according to the treatment scheme (hydroxychloroquine, hydroxychloroquine+oseltamivir, and favipiravir) applied due to COVID-19. Patients were evaluated according to where they were followed (home isolation or hospitalization).

Evaluation of olfactory functions with BSIT

The BSIT (Sensonics, Inc, Haddon Heights, NJ), which contains 12 different scents, is a reliable test that must be smelled after scratching [7]. The total score in the test with four-choice questions is 12. While values of eight and/or below are accepted as olfactory dysfunction, values of nine and/or above are considered normal [8]. According to BSIT values, patients were divided into severe (0 to two), moderate (three to five), and mild (six to eight) olfactory dysfunction [8].

Statistical analysis

SPSS (IBM Corp. Released 2016. IBM SPSS Statistics for Windows, Version 24.0. Armonk, NY: IBM Corp) was used for statistical analysis. Descriptive statistics related to continuous data were stated as mean ± standard deviation. The statistical value of p<0.05 was considered significant. Kolmogorov-Smirnov test and Shapiro-Wilk test were used as normality tests. While the saturation values at the time of admission and the last saturation values were not normally distributed, the other values were normally distributed. BSIT scores were compared with the paired samples t-test. Wilcoxon rank test was used to compare the first and last saturation values. Correlation analyses were performed with the Pearson correlation test.

## Results

Demographic features and laboratory findings of the study, which included 64 patients, are given in Table 1. There was no statistical difference observed between age and gender distribution, fever, complaints at admission, the place where the patients were followed, and the treatment scheme (p>0.05). The distribution of patients according to clinical features is given in Table 2. There was no significant difference seen between the first and after 14 days of laboratory values of the patients (p>0.05). In addition, no correlation was found between laboratory findings and BSIT scores (p>0.05).

**Table 1:** Demographic features and laboratory findings (Mean (±SD)) *: Paired sample t-test was used. SD: standard deviation; WBC: white blood cell; CRP: C-reactive protein.

		Age, Gender and Laboratory Findings
Age (Mean (±SD))		44.09±15.29
Gender (n (%))	Female	26 (40.6%)
	Male	38 (59.4%)
WBC (x1000 uL)	Initial values	7.52±1.21
	14th day values	7.55±1.09
	p values	0.36^*^
Hemoglobin (g/dL)	Initial values	13.15±1.56
	14th day values	13.14±1.44
	p values	0.23^*^
Platelet (x1000 uL)	Initial values	242.69±51.25
	14th day values	240.33±49.14
	p values	0.44^*^
Ferritin (µg/L)	Initial values	297.20±89.41
	14th day values	298.32±96.23
	p values	0.49^*^
CRP (mg/L)	Initial values	3.15±0.42
	14th day values	3.14±1.02
	p values	0.30^*^

**Table 2:** Clinical features

Clinical Features		Distribution of Patients (n=64) (%)
Fever	Yes (37.5 °C and above)	39 (60.9%)
	No (37.5 °C and below)	25 (39.1%)
Treatment Scheme	Hydroxychloroquine	21 (32.8%)
	Hydroxychloroquine+Oseltamivir	18 (28.1%)
	Favipiravir	25 (39.1%)
Follow-up Place	Followed at Home	48 (75.0%)
	Followed at Hospital	16 (25.0%)
Complaints at Admission	Fever (37.5 °C and above)	23 (35.9%)
	Cough	7 (10.9%)
	Fever+Cough	8 (12.5%)
	Fever+Cough+Dyspnea	7 (10.9%)
	Cough+Dyspnea	6 (9.4%)
	Runny Nose	7 (10.9%)
	Olfactory Dysfunction	6 (9.4%)

BMI, SpO2 levels, and BSIT scores are given in Table 3. Blood oxygen saturation values at first admission and BMI values were negatively correlated (p=0.03). No correlation was found between BMI values, after 14 days of SpO2 levels and the change in saturation (p>0.05). When BMI and olfactory function were evaluated together, no correlation was found between improvement in olfactory functions and BMI (p=0.55). However, a negative correlation was found between BMI and BSIT values performed at the first admission and when PCR became negative (p=0.03 and p=0.04). No correlation was found between BMI and fever and the place where the patients were followed (p>0.05).

**Table 3:** BMI, SpO2 levels, and BSIT values *: Paired sample t-test was used. ¥: Wilcoxon rank test was used. SD: standard deviation; BMI: body mass index; SpO2: blood oxygen saturation; BSIT: brief smell identification test.

		BMI, SpO2 and BSIT Values
BMI (cm/kg) (Mean (±SD))		26.86±3.60
SpO2 Levels (%)	Initial values	96.34±3.83
	14th day values	98.75±0.71
	p values	<0.001^¥^
BSIT	Initial values	5.60±2.53
	14th day values	7.15±2.02
	p values	<0.001^*^

The first SpO2 levels of the patients were found to be significantly lower than the 14th day Sp02 levels (p<0.001). No correlation was found between the improvement in Sp02 levels and the improvement in olfactory functions (p>0.05). A positive correlation was found between Sp02 levels at admission and the BSIT values at admission (p=0.02). The improvement in Sp02 levels in patients with high fever was found to be significantly less than those without fever (p=0.03). In patients who were followed up at home, the improvement in Sp02 levels was found to be significantly higher than those followed in the hospital (p<0.001).

Olfactory function outcomes

Olfactory dysfunction (BSIT score ≤8) was detected in 57 (89%) of the patients, while six patients had severe, 23 patients had moderate, and 28 patients had mild olfactory dysfunction. According to the BSIT scores on the 14th day, olfactory dysfunction was observed in 48 (75%) of the patients. According to the final BSIT scores, olfactory dysfunction was detected as severe in one patient, moderate in 12 patients, and mild in 35 patients. A significant difference was found between the BSIT values at admission and the BSIT values performed on the 14th day (p<0.001). Significant improvements in olfactory function were demonstrated 14 days after the first test. In six (9.4%) patients olfactory dysfunction was observed as the initial complaint (Table 2). There was no significant difference between patients with high fever and patients without fever in terms of improvement of olfactory functions (p=0.73). No correlation was found between the patient's complaints at admission and the improvement in olfactory functions (p=0.63). There was no correlation between the different treatment options and the improvement in olfactory functions (p=0.26). There was no significant difference in the improvement of olfactory functions between the patients followed at home and those followed in the hospital (p=0.85).

## Discussion

With COVID-19 spreading all over the world, the number of patients with olfactory dysfunction is increasing rapidly. Although objective and subjective tests are used to detect olfactory dysfunction, significant inconsistencies have been reported between both test groups [9]. In our study, BSIT, which is an objective and reliable test with 12 different scent options, that can be performed in a short time, was used.

The olfactory disorder, which can be the distinguishing and first symptom of COVID-19 disease caused by SARS-CoV-2, ranges from 33.9% to 85.6% [5]. In a retrospective study conducted by Mao et al. in the early days of the COVID-19 outbreak in Wuhan, China, the most common symptoms of COVID-19 were reported as taste (5.6%) and smell (5.1%) disorders [10]. However, as COVID-19 spread and related studies increased, the frequency of olfactory disorders was reported to be higher [11-13]. Petrocelli et al. reported olfactory dysfunction in 63.3% of 300 patients in their study [14]. In addition, anosmia was found in 47% of the patients and hyposmia in 16.3% [14]. According to the BSIT score, Zhu et al. detected olfactory dysfunction in 22 (23.2%) of 95 patients [15]. In our study, a BSIT score of eight or less was found in 57 (89%) of 64 patients and it was evaluated as olfactory dysfunction.

Uğurlu et al., in a study conducted with 42 patients, found that the first complaint was olfactory dysfunction in 40% of the patients and the mean BSIT score was 5.2±2.2. They also reported 16.7% severe, 31% moderate, and 52.4% mild olfactory dysfunction [11]. After three months of follow-up, the BSIT score was found to be 9.9±1.8 and it was reported that it increased significantly [11]. In another study, a significant difference was found between the values of BSIT scores at the first admission and 14th weeks (5.29±2.02 and 8.29±2.40) [15]. In our study, unlike other studies, we showed short-term clinical and olfactory function changes caused by COVID-19. The BSIT values on the first and 14th days were 5.60±2.53 and 7.15±2.02, while a significant difference was found.

Catton et al. in 2022 reported in their study the mean BMI was 26.81 in COVID-19 patients who had a loss of smell for less than 28 days [16]. Algahtani et al. found the mean BMI as 26.64 in their study with 808 COVID-19 patients and they correlated BMI values and anosmia scores that developed within 14 days [17]. In our study, the BMI score was found to be 26.86±3.60 and no correlation was found between BMI and improvement in olfactory functions. However, BSIT values at the first admission and on the 14th day were negatively correlated with BMI. In our study, no correlation was found between fever, the place of follow-up, and BMI. In a study with 300 patients, Oda et al. reported that BMI values were significantly higher in patients with decreased SpO2 [18]. In our study, a negative correlation was found between SpO2 levels at admission and BMI values, but no correlation was found between the improvement in SpO2 levels and BMI.

In a study of 727 patients Ashrafi et al. found a significant difference in terms of oxygen saturation between patients with neurological symptoms such as olfactory dysfunction and those without neurological complaints [19]. In our study, low SpO2 levels at the first admission were associated with low BSIT scores. However, no correlation was found between the improvement in SpO2 levels and the improvement in olfactory functions. In the study of Singer-Cornelius et al., high fever was found in 26.8% of patients with olfactory dysfunction [20]. In our study, fever was detected in 60.9% of the patients. However, no relationship was found between olfactory dysfunction and high fever.

The small sample size and inability to work with intensive care patients limit the present results. Future studies should involve longer periods of follow-up, more objective evaluation of olfactory function testing, larger patient series, and more clinical features.

## Conclusions

Many studies have aimed to evaluate olfactory functions in the medium and long-term duration. In our study, a high rate of 89% olfactory dysfunction was found in the 14-day follow-up period. Low saturation values at first admission were associated with low BSIT scores. As a result, the negative effect of COVID-19 disease on olfactory functions has been shown, even in a short follow-up period.
